# B‐Type Natriuretic Peptide as a Marker of Subclinical Heart Disease in a High‐Burden Emergency Department Population With Sustained Asymptomatic Hypertension

**DOI:** 10.1155/ijhy/9951414

**Published:** 2025-12-12

**Authors:** Kimberly Souffront, Bret P. Nelson, Megan Lukas, Hans Reyes Garay, Claire Shubeck, Lauren Gordon, George T. Loo, Aleksandra Degtyar, Marcee Wilder, Hanisah Iddrisu, Bernice Coleman, Lynne D. Richardson

**Affiliations:** ^1^ Icahn School of Medicine at Mount Sinai, New York, New York, USA, mountsinai.org; ^2^ Mount Sinai Health System, New York, New York, USA, mountsinai.org; ^3^ Department of Cardiology, Mount Sinai Morningside, New York, New York, USA; ^4^ School of Medicine, Tufts University, Boston, Massachusetts, USA, tufts.edu; ^5^ The College at Brown University, Providence, Rhode Island, USA; ^6^ Cedars-Sinai Medical Center, Los Angeles, California, USA, cedars-sinai.edu

## Abstract

B‐Type natriuretic peptide (BNP) levels below contemporary thresholds for diagnosing congestive heart failure are associated with subclinical heart disease (SHD) and adverse cardiovascular outcomes in community patients with uncontrolled asymptomatic hypertension. This study aimed to determine the accuracy of BNP for detecting SHD in emergency patients with sustained asymptomatic hypertension, where SHD is universally prevalent. Conducted at two urban academic emergency departments within a seven‐hospital healthcare organization, this proof‐of‐concept study included adults with sustained asymptomatic hypertension (initial BP ≥ 160/100 mmHg and second BP ≥ 140/90 mmHg), excluding those with congestive heart failure, renal insufficiency, atrial fibrillation, pregnancy, incarceration, cognitive impairment, and symptomatic hypertension. Enrolled patients underwent bedside echocardiograms, BNP lab tests, and electrocardiograms. All 78 patients (100%) had SHD. The cohort was predominantly female (55.1%), middle‐aged (mean age: 52 ± 15.2 years), with Class I obesity (mean BMI: 32.3 ± 8.3) and a high prevalence of hypertension history (55.1%). Common findings included left ventricular hypertrophy (86%), diastolic dysfunction (70.5%), and left ventricular systolic dysfunction (12.2%). The BNP lab test accurately detected SHD in nearly 60% of patients, with a Type II error rate of approximately 40%. In this proof‐of‐concept study, BNP underperformed in a cohort with universally present SHD, suggesting that sole reliance on BNP may lead to missed opportunities for early intervention.

## 1. Introduction

Hypertension is the leading risk factor for morbidity and mortality worldwide and is prevalent among patients in United States emergency departments (EDs) [1–4]. While often dismissed as transient or anxiety‐related, elevated blood pressure in the ED reliably identifies chronic uncontrolled hypertension [4–8]. These patients face a high burden of subclinical heart disease (SHD) [9–12], a precursor to overt heart failure that typically goes undetected and untreated. Despite this, follow‐up guidance for asymptomatic hypertension is rarely provided by emergency clinicians, representing a missed opportunity for early intervention [4, 14–17]. A point‐of‐care marker of SHD for ED patients with sustained asymptomatic hypertension may help bridge this gap and improve vigilance with follow‐up to care.

B‐Type natriuretic peptide (BNP) has been associated with cardiac remodeling and adverse outcomes [18–20], even below thresholds used to diagnose heart failure. However, its potential as a point‐of‐care marker for detecting SHD in the ED remains largely uncharacterized [21, 22]. Prior studies generally support a correlation between BNP/NT‐BNP and early hypertensive heart disease, and, in some cases, suppression of peptide levels despite underlying cardiac stress [23, 24]. For example, Belluardo et al. [23] studied NT‐BNP in an Italian outpatient cohort, while Macheret et al. [24] evaluated BNP levels in a community‐based sample from Olmsted County, Minnesota, both suggesting that BNP may be an unreliable signal in the early phases of hypertensive heart disease [25]. However, race and ethnicity were not reported in these cohorts, and their predominantly outpatient, nondiverse populations differ substantially from the ED setting, where patients frequently present with chronic, uncontrolled hypertension and higher cardiovascular risk [9–12].

This proof‐of‐concept study explored the utility of BNP in a multisite cohort of ED patients with sustained asymptomatic hypertension, a high‐risk population in which cardiovascular disease is common but often unrecognized. Rather than establishing generalizable diagnostic thresholds, we sought to determine whether BNP could serve as a practical signal of early cardiac involvement in this context, describe its distribution in this population, and explore factors that may influence its clinical utility.

## 2. Methods

### 2.1. Study Design, Sample, and Setting

This proof‐of‐concept study recruited patients from two urban academic EDs that serve diverse communities in New York City. English‐ or Spanish‐speaking adults (≥ 18 years old) patients pending discharge from the ED, whose initial BP was ≥ 160/100 mmHgand second BP ≥ 140/90 mmHg, were included in the study. Patients with renal insufficiency, congestive heart failure, or atrial fibrillation, or who were pregnant, prisoners, cognitively unable to provide informed consent, or experiencing symptoms of hypertension such as chest pain, paresthesia, or shortness of breath, were excluded. These symptoms have previously been identified as symptomatic hypertension in emergency care [26].

### 2.2. Data Collection

Data collection began after IRB approval. Three days a week, a research coordinator recruited and enrolled eligible ED patients. After obtaining consent and physician agreement, data were recorded via REDCap [27, 28]. If not previously performed, an electrocardiogram (EKG) was performed. A trained team member conducted a bedside echocardiogram in under 10 min. The ED provider placed a BNP order in the electronic health record, and the ED nurse obtained and sent the lab as per the standard procedure. Participants received $50 for their involvement.

### 2.3. Measures

#### 2.3.1. Patient Characteristics

Gender, race/ethnicity, age, primary care provider status, body mass index (BMI), and history of smoking, hypertension, and diabetes were recorded. BMI was calculated only when both height and weight were available in the electronic health record. Data were entered into REDCap [27, 28] by two team members for accuracy, with discrepancies resolved by the principal investigator (KS).

#### 2.3.2. Echocardiogram

Following the American College of Cardiology, American Heart Association, Heart Failure Society of America, and Levy et al. [10, 27, 28], three endpoints were assessed [29] as follows:

1. Left ventricular hypertrophy (LVH): Present if left ventricular septal thickness ≥ 1.1 cm; absent if < 1.1 cm. Measured at end diastole in the parasternal long view.
2. Left ventricular systolic dysfunction: Ejection fraction was categorized as normal (≥ 55%) or abnormal (< 55%).
3. Diastolic dysfunction: Evaluated using the *E*/*e*’ ratio to estimate left atrial pressure. Diastolic dysfunction was present if *E*/*e*’ (septal) ≥ 15, *E*/*e*’(lateral) ≥ 12, septal *e*’ < 8 cm/sec, or lateral *e*’ < 10 cm/sec.

#### 2.3.3. EKG

LVH was diagnosed using the Cornell voltage criteria (sum of S wave in lead V3 and R wave in lead aVL > 20 mm in women and > 23 mm in men). Heart rate, PR interval, and QRS interval were also recorded from the EKG [30].

#### 2.3.4. BNP

Abnormal BNP levels were defined as > 20 pg/mL in men and > 23.3 pg/mL in women based on the Framingham study [18, 31]. For the last 20 participants, BNP levels were measured using the Triage Meter Pro BNP Panel, a point‐of‐care fluorescence immunoassay. These participants were re‐consented for another aim of the study [32]. All other tests were conducted in the hospital laboratory, where data were collected. The Triage Meter Pro BNP test, with CLIA waiver and FDA approval, has high specificity compared to traditional assays [32]. Blood samples were collected using EDTA tubes with aprotinin (lavender top).

### 2.4. Sample Size Calculation

Literature indicates that the point prevalence of SHD among ED population with uncontrolled HTN ranges from 40% to 90% [9, 10, 12]. Assuming a 95% confidence interval and 0.05 precision, we calculated that a sample size of 76 subjects would be needed to achieve 97% sensitivity and 90% specificity, assuming a 60% SHD point prevalence. We then enrolled 76 participants to meet the target sample size.

### 2.5. Data Analysis

Data were exported from REDCap to SAS Version 9.4 for analysis [27, 28, 33]. Percentages or means (*M*) and standard deviations (SDs) were calculated for patient characteristics. SHD was categorized as abnormal if LVH, diastolic dysfunction, systolic dysfunction, or any combination were present; otherwise, it was categorized as normal. Fisher’s exact test assessed the relationship between patient characteristics and abnormal BNP lab test (yes/no). We also aimed to describe the sensitivity, specificity, positive predictive value (PPV), and negative predictive value (NPV) of BNP for detecting SHD.

This was a descriptive exploratory analysis designed to examine whether BNP could serve as a practical signal for SHD in a high‐risk ED population. Given that SHD was universally present in this cohort, traditional diagnostic accuracy metrics (e.g., sensitivity, specificity, and PPV) could not be meaningfully assessed. Our aim was not to establish diagnostic thresholds but to describe BNP distribution in this population and to explore potential factors that may influence its clinical utility.

## 3. Results

A total of 84 participants were enrolled. Data from six participants were missing, leaving 78 participants for analysis. The majority were female (*n* = 43, 55.1%), middle‐aged (μ = 52, SD = 15.2), Hispanic (*n* = 41, 57.8%), and self‐identified as Black (*n* = 29, 38.7%) or unknown race/ethnicity (*n* = 28, 37.3%). The mean initial systolic BP was 172.4 (SD = 14.6) and diastolic BP was 100.5 (SD = 13.6). The mean second systolic BP was 162.6 (SD = 19.0) and the diastolic BP was 95.0 (SD = 15.5). Over half reported known hypertension (55.1%) and having a primary care provider (56.4%). Approximately one‐third were smokers (*n* = 25, 32.1%). The mean BMI was 32.3 (SD = 8.3), indicating Class 1 Obesity. Clinical impressions included orthopedic (*n* = 33, 42.3%), gastrointestinal (*n* = 20, 25.6%), genitourinary (*n* = 5, 6.4%), pulmonary (*n* = 4, 5.1%), neurological (*n* = 4, 5.1%), dermatological (*n* = 11, 14.1%), and chronic disease issues (*n* = 1, 1.3%), excluding hypertension (Table 1).

**Table 1:** Patient characteristics of the final sample.

**Patient characteristics**	***N* = 78**

Age, μ (SD)	52 (15.2)
Gender, *n* (%)	
Female	43 (55.1)
Male	35 (44.9)
Race, *n* (%)	
Caucasian/White	18 (24)
African American/Black	29 (38.7)
Other or unknown	28 (37.3)
Ethnicity, *n* (%)	
Not Hispanic/Latino	30 (42.1)
Hispanic/Latino	41 (57.8)
Missing	7 (0.1)
Blood pressure, μ mm Hg (SD)	
1^st^ Systolic blood pressure, μ (SD)	172.4 (14.6)
1^st^ Diastolic blood pressure, μ (SD)	100.5 (13.6)
2^nd^ Systolic blood pressure, μ (SD)	162.6 (19.0)
2^nd^ Diastolic blood pressure, μ (SD)	95.0 (15.5)
Hypertension history, yes	43 (55.1)
Took medicine in the last 3 months	26 (26.7)
Smoker, yes	25 (32.1)
Diabetes mellitus history, yes	19 (24.3)
Body mass index, μ (SD)	32.3 (8.3)
Primary care physician, yes	44 (56.4)
Missing BMI *n* = 23	

Abbreviations: BMI, body mass index; mm Hg, millimeters of mercury.

### 3.1. Echocardiogram

All participants (*n* = 78) showed evidence of SHD, corresponding to Stage B heart failure (Table 2). Most had LVH (*n* = 67, 86%), diastolic dysfunction (*n* = 55, 70.5%), and systolic dysfunction (*n* = 10, 12.2%). No significant differences were found between SHD and gender, age, race, ethnicity, or hypertension history (Table 3).

**Table 2:** Abnormal brain natriuretic peptide and associations.

Patient characteristics	Abnormal BNP *n* = 44 (58%)	*p* value	Odds ratio (95% confidence interval)
BNP, mean (SD)	51.1 (67.6)		
Gender			
Male, *n* (%)			0.5 (0.2–1.3)
Female, *n* (%)	27 (36)	0.23	
Race			
Black or African American, *n* (%)	16 (21.3)	0.45	
White, *n* (%)	13 (17.3)		
Other or missing, *n* (%)	15 (20)		
Ethnicity			
Hispanic, *n* (%)	22 (29.3)	0.5	
Hypertension history – yes, *n* (%)	22 (29)	0.6	0.7 (0.3–1.8)
Smoking history ‐ yes, *n* (%)	12 (16)	0.3	0.6 (0.2–1.6)
Diabetes history – yes, *n* (%)	11 (14.3)	0.6	1.5 (0.5–4.6)

Abbreviation: BNP, B‐type natriuretic peptide.

**Table 3:** SHD and BNP results.

**Subclinical heart disease and type**	**SHD *N* = 78 (100%)**	***p* value**	**Odds ratio (OR) (95% confidence interval)**

Left ventricular hypertrophy	67 (86)	0.74	[1.5 (0.40–5.6)]
Diastolic dysfunction	55 (70.5)	0.61	[1.4 (0.52–3.76)]
Left ventricular systolic dysfunction	10 (12.2)	1.0	[0.7 (0.2–3.0)]

### 3.2. EKG

Few participants (*n* = 4, 5.1%) had an abnormal EKG, with a mean heart rate of 77 beats/min. No significant differences were found between abnormal EKG and gender, age, race, ethnicity, or hypertension history.

### 3.3. BNP

The mean BNP level was 51.1 pg/mL (SD = 67.6), with 58% (*n* = 44) having an abnormal BNP. No significant differences were found between abnormal BNP and gender, age, race, ethnicity, or hypertension history (Table 3).

### 3.4. Sensitivity

The BNP test detected SHD in nearly 60% of patients with sustained asymptomatic hypertension, with a Type II error rate of 40%. As there were no disease‐free participants, specificity and predictive values were not calculated.

## 4. Discussion

In this proof‐of‐concept study, BNP showed limited ability to detect SHD in a cohort of ED patients with sustained asymptomatic hypertension, even though structural cardiac abnormalities were universally present [11]. Our findings are consistent with prior reports by Belluardo et al. [23] and Macheret et al. [24], who demonstrated suppressed BNP response in early hypertension, though their cohorts were not diverse and non‐ED based [25]. By observing similar patterns in a racially and ethnically diverse, urban ED population, our results extend this evidence to a distinct clinical and demographic setting. Together, our findings add to prior work by suggesting that BNP’s ability to detect SHD may not consistently replicate findings from larger outpatient studies such as PROBE‐HF [19] and PREDICTOR [34], which were also limited in diversity. Although our study was not powered or designed to establish diagnostic accuracy, these studies still highlight the need for research in diverse groups such as ours [34].

Our cohort exhibited a high prevalence of Class 1 obesity, potentially contributing to lower BNP levels. Although we observed a high mean BMI in our cohort, we were unable to consistently assess waist circumference or formally correlate BNP and BMI due to missing data. However, prior research has shown that BNP levels are often suppressed in individuals with visceral obesity and hypertension, which may explain the observed underperformance. Multiple studies across Western and Asian populations have demonstrated an inverse relationship between BMI and circulating BNP levels, independent of age, sex, and cardiac structure [35–37]. This suppression has been observed even in the absence of heart failure and across different cultural contexts, suggesting a physiological effect of adiposity rather than lifestyle factors. Our findings highlight the need for biomarkers validated across diverse body compositions and clinical contexts.

There is also evidence that BNP underperforms in patients with hypertension, which was recorded in 55.1% of our cohorts’ PMH, although all were hypertensive upon enrollment. For example, BNP levels were reduced in patients with Stage 1 hypertension compared to controls, and only increased in later stages of hypertension, independent of LVH presence in one prospective observational study of 78 patients with hypertension and 28 age‐ and sex‐matched controls. In addition, Macheret et al. [24] found no consistent difference in BNP across blood pressure categories and suggested a possible deficiency in early hypertension [24]. These findings align with the hypothesis that BNP suppression in early hypertension may reflect impaired cardiac endocrine response or increased peptide clearance in obese individuals [25].

Antihypertensive medications may have also led to falsely low BNP levels. More than half of our population had a hypertension history, with many taking medications such as ACE inhibitors, beta blockers, and angiotensin receptor antagonists, all of which may blunt natriuretic peptide release. Prior studies have also demonstrated that BNP levels tend to be lower among Black and Mexican American adults compared to White adults, independent of cardiometabolic risk [20]. This is significant in light of the demographic composition of our cohort. However, race and ethnicity should not be interpreted as genetic proxies; instead, structural, environmental, and physiologic factors likely contribute to these observed differences [20]. These findings underscore the importance of validating biomarkers in diverse populations. Further research is warranted to assess BNP performance in normotensive or early‐stage hypertensive patients and to identify alternative or adjunctive screening strategies for SHD in emergency care settings.

## 5. Limitations

Our study suggests that sustained asymptomatic hypertension (e.g., blood pressure) may be the most reliable and clinically actionable indicator of SHD. Moreover, results should be interpreted within the context of a hypothesis‐generating, descriptive design, and not a diagnostic validation study. In addition, waist circumference, a relevant marker of visceral adiposity, was not consistently collected due to practical constraints of the ED setting. Since BMI data were available for only a subset of patients (*n* = 25), a correlation analysis between BNP and BMI was not performed. This remains an important direction for future investigation [26].

## 6. Conclusion

In this proof‐of‐concept study, BNP underperformed as a marker for detecting SHD in ED patients with sustained asymptomatic hypertension, despite SHD being universally present. Factors such as obesity, hypertension stage, medication use, and demographic differences may influence biomarker performance and should be considered. Given the high prevalence of SHD, sustained asymptomatic hypertension itself may serve as the most actionable indicator for initiating follow‐up and further evaluation. Future research should validate alternative or adjunctive markers in diverse ED populations to support earlier detection and intervention.

## Conflicts of Interest

The authors declare no conflicts of interest.

## Funding

This study was funded by the Gordon and Betty Moore Foundation through the Betty Irene Moore Fellowship for Nurse Leaders and Innovators (GBMF9048) (K.S.), the National Institutes of Health (NIH)/National Heart, Lung, and Blood Institute (5T32HL160513) (A.D.), and the NIH/National Cancer Institute (5U54CA267776) (M.W.).

## Data Availability

The data that support the findings of this study are available from the corresponding author upon reasonable request.
